# Hemophagocytic lymphohistiocytosis after COVID-19 vaccination

**DOI:** 10.1186/s13045-021-01100-7

**Published:** 2021-06-04

**Authors:** Liang V. Tang, Yu Hu

**Affiliations:** Institute of Hematology, Union Hospital, Tongji Medical College, Huazhong University of Science and Technology, Wuhan, China

**Keywords:** Hemophagocytic lymphohistiocytosis, SARS-CoV-2 vaccine, Epstein–Barr virus, Coagulopathy, COVID-19

## Abstract

Cases of thrombotic thrombocytopenia induced by coronavirus disease 2019 (COVID-19) vaccines have been reported recently. Herein, we describe the first case of another critical disorder, hemophagocytic lymphohistiocytosis (HLH), in a healthy individual after COVID-19 vaccination. A 43-year-old Chinese farmer developed malaise, vomiting, and persistent high fever (up to 39.7 °C) shortly after receiving the first dose of the inactivated SARS-CoV-2 vaccine. The initial evaluation showed pancytopenia (neutrophil count, 0.70 × 10^9^/L; hemoglobin, 113 g/L; platelet, 27 × 10^9^/L), elevated triglyceride (2.43 mmol/L), and decreased fibrinogen (1.41 g/L). Further tests showed high serum ferritin levels (8140.4 μg/L), low NK cell cytotoxicity (50.13%–60.83%), and positive tests for Epstein–Barr virus (EBV) DNA. Hemophagocytosis was observed in the bone marrow. Therefore, HLH was confirmed, and dexamethasone acetate (10 mg/day) was immediately prescribed without etoposide. Signs and abnormal laboratory results resolved gradually, and the patient was discharged. HLH is a life-threatening hyperinflammatory syndrome caused by aberrantly activated macrophages and cytotoxic T cells, which may rapidly progress to terminal multiple organ failure. In this case, HLH was induced by the COVID-19 vaccination immuno-stimulation on a chronic EBV infection background. This report indicates that it is crucial to exclude the presence of active EBV infection or other common viruses before COVID-19 vaccination.

**To the editor**

Cases of thrombotic thrombocytopenia induced by coronavirus disease 2019 (COVID-19) vaccines have been reported recently [1–3]. Herein, we describe the first case of another critical disorder, hemophagocytic lymphohistiocytosis (HLH), in a healthy person after COVID-19 vaccination.

A 43-year-old Chinese female farmer developed malaise, vomiting, and a fever of 37.6 °C shortly after receiving the first dose of the inactivated SARS-CoV-2 vaccine. One day later, she presented with a persistent high fever (up to 39.7 °C). Treatment with antibiotics and nonsteroidal anti-inflammatory drugs was ineffective. On the eighth day, the patient was admitted to our hospital. The initial evaluation showed pancytopenia (neutrophil count, 0.70 × 10^9^/L; hemoglobin, 113 g/L; platelet, 27 × 10^9^/L), elevated triglyceride (2.43 mmol/L), decreased fibrinogen (1.41 g/L), and increased transaminase (AST 254 U/L) and lactate dehydrogenase (1033 U/L) levels. Further tests showed a high serum ferritin level (8140.4 μg/L), low NK cell cytotoxicity (50.13%–60.83%), positive tests for Epstein–Barr virus DNA (EBV, 2.47 × 10^5^ copy/ml in whole blood and 824 copy/ml in plasma), and negative tests for SARS-CoV-2 RNA and IgM/IgG antibodies. Hemophagocytosis was observed in the bone marrow. The results of the laboratory and imaging tests are summarized in Table 1. Therefore, HLH was confirmed based on both the HLH-2004 diagnostic criteria (fulfilling six out of the eight criteria) and the HLH-probability calculator (HScore, up to 261). According to “the recommendations for the management of HLH” [4], dexamethasone acetate (10 mg/day) was immediately prescribed. The signs and abnormal laboratory results resolved gradually without the addition of a cytotoxic drug (etoposide), and the patient was discharged 17 days later (Fig. 1). The glucocorticoid dose was tapered carefully, and follow-up is still ongoing.

**Table 1:** Laboratory and imaging tests on admission

Tests on admission	Results	Normal ranges
Complete blood count		
White blood cell (10^9^/L)	**1.26**	3.5–9.5
Neutrophil count (10^9^/L)	**0.70**	1.8–6.3
Lymphocyte count (10^9^/L)	**0.49**	1.1–3.2
Hemoglobin (g/L)	**113**	130–175
Platelet (10^9^/L)	**27**	125–350
Reticulocyte count (10^12^/L)	0.04	0.024–0.084
Coagulation		
APTT (s)	**61.6**	28.0–43.5
PT (s)	13.9	11.0–16.0
Thrombin time (s)	**39.9**	14.0–21.0
Fibrinogen (g/L)	**1.41**	2.0–4.0
D-Dimer (mg/L)	**6.80**	< 0.5
Hepatic and renal function		
ALT (U/L)	**141**	5–35
AST (U/L)	**254**	8–40
Total bilirubin (μmol/L)	7.1	3–22
Direct bilirubin (μmol/L)	0.0	0–5
Lactate dehydrogenase (U/L)	**1033**	109–245
Albumin (g/L)	**27.2**	35–50
Globulin (g/L)	27.1	23–32
Blood urea nitrogen (mmol/L)	**2.43**	2.5–6.1
Creatinine (μmol/L)	54.0	46.0–92.0
Fasting lipid		
Triglycerides (mmol/L)	**2.43**	< 1.7
Total cholesterol (mmol/L)	2.44	< 5.2
HDL-C (mmol/L)	**0.45**	1.29–1.55
LDL-C (mmol/L)	**1.05**	2.7–3.1
Lymphocyte subsets		
CD4^+^ T cells (%)	**17.67**	25.34–51.37
CD8^+^ T cells (%)	**71.72**	14.23–38.95
NK cells (%)	3.78	3.33–30.47
NK cell cytotoxicity-granzyme (%)	**50.13%**	> 78%
NK cell cytotoxicity-perforin (%)	**60.83%**	> 84%
Inflammatory factors		
Ferritin (μg/L)	**8140.4**	4.6–204.0
hsCRP (mg/L)	**10.75**	0–5
sCD25 (pg/ml)	**204.99**	3.71–16.05
IL-1β (pg/ml)	**8.21**	< 3.40
IL-2 (pg/ml)	0.76	< 6.64
IL-4 (pg/ml)	1.67	< 4.19
IL-6 (pg/ml)	**17.55**	< 5.30
IL-8 (pg/ml)	**90.41**	< 15.71
IL-10 (pg/ml)	**18.45**	< 4.91
IL-12p70 (pg/ml)	0.00	< 10.18
IL-17A (pg/ml)	2.33	< 4.74
IL-17F (pg/ml)	0.19	< 4.66
IL-22 (pg/ml)	0.4	< 3.64
TNF-α (pg/ml)	3.96	< 4.50
TNF-β (pg/ml)	**17.63**	< 2.54
INF-γ (pg/ml)	1.86	< 4.43
Complement 3 (g/L)	**0.625**	0.790–1.520
Complement 4 (g/L)	0.182	0.160–0.380
Virus		
Whole blood EBV DNA (copy/ml)	**2.47 × 10**^**5**^	< 400.00
Plasma EBV DNA (copy/ml)	**824**	< 400.00
EB-VCA IgA (AU/ml)	0.79	< 4.00
EB-VCA IgM (AU/ml)	0.45	< 3.00
EB-VCA IgG (AU/ml)	**> 50.00**	< 2.00
EB-VEA IgA (AU/ml)	0.44	< 3.00
EB-VEA IgG (AU/ml)	0.36	< 2.00
EB-VNA IgG (AU/ml)	**> 50.00**	< 2.00
Whole blood CMV DNA (copy/ml)	< 400.00	< 400.00
Plasma CMV DNA (copy/ml)	< 400.00	< 400.00
SARS-CoV-2 RNA	Negative	Negative
Anti-SARS-CoV-2 IgM	Negative	Negative
Anti-SARS-CoV-2 IgG	Negative	Negative
Hepatitis B surface antigen	Negative	Negative
Hepatitis C virus antibody	Negative	Negative
HIV antibody	Negative	Negative
Anti-nuclear antibody spectrum	Negative	Negative
Hemophagocytosis in bone marrow	**Positive**	Negative
Superficial lymph nodes (Ultrasonography)	Negative imaging	Negative imaging
Color Doppler echocardiography	Negative imaging	Negative imaging
Chest CT scan	Negative imaging	Negative imaging
Abdominal CT scan	Negative imaging	Negative imaging
Pelvic CT scan	Negative imaging	Negative imaging
HLH-associated genes	Wild type	Wild type
HLH-2004 diagnostic criteria	**6 of the 8 criteria**	Cut-off: 5 of the 8 criteria
HLH-probability calculator (HScore)	**261**	Cut-off: 169

Abnormal results are shown in BOLD

APTT: activated partial thromboplastin time; PT: prothrombin time; EB-VCA: Epstein–Barr virus capsid antibody; EB-VEA: Epstein–Barr virus early antibody; EB-VNA: Epstein–Barr virus nuclear antibody; CMV: Cytomegalovirus; HLH: Hemophagocytic lymphohistiocytosis; HLH-associated genes: *AP3B1, ARF6, BLOC1S6, CD27, CARD11, CORO1A, CTPS1, GNLY, GZMB, IL2RG, ITK, LAMP1, LYST, MAGT1, MCM4, PRF1, PIK3CD, PRKCD, RAB27A, SH2D1A, SRGN, STX11, STK4, STXBP2, TCN2, UNC13D, CTPS1, AND XIAP*

**Fig. 1:**
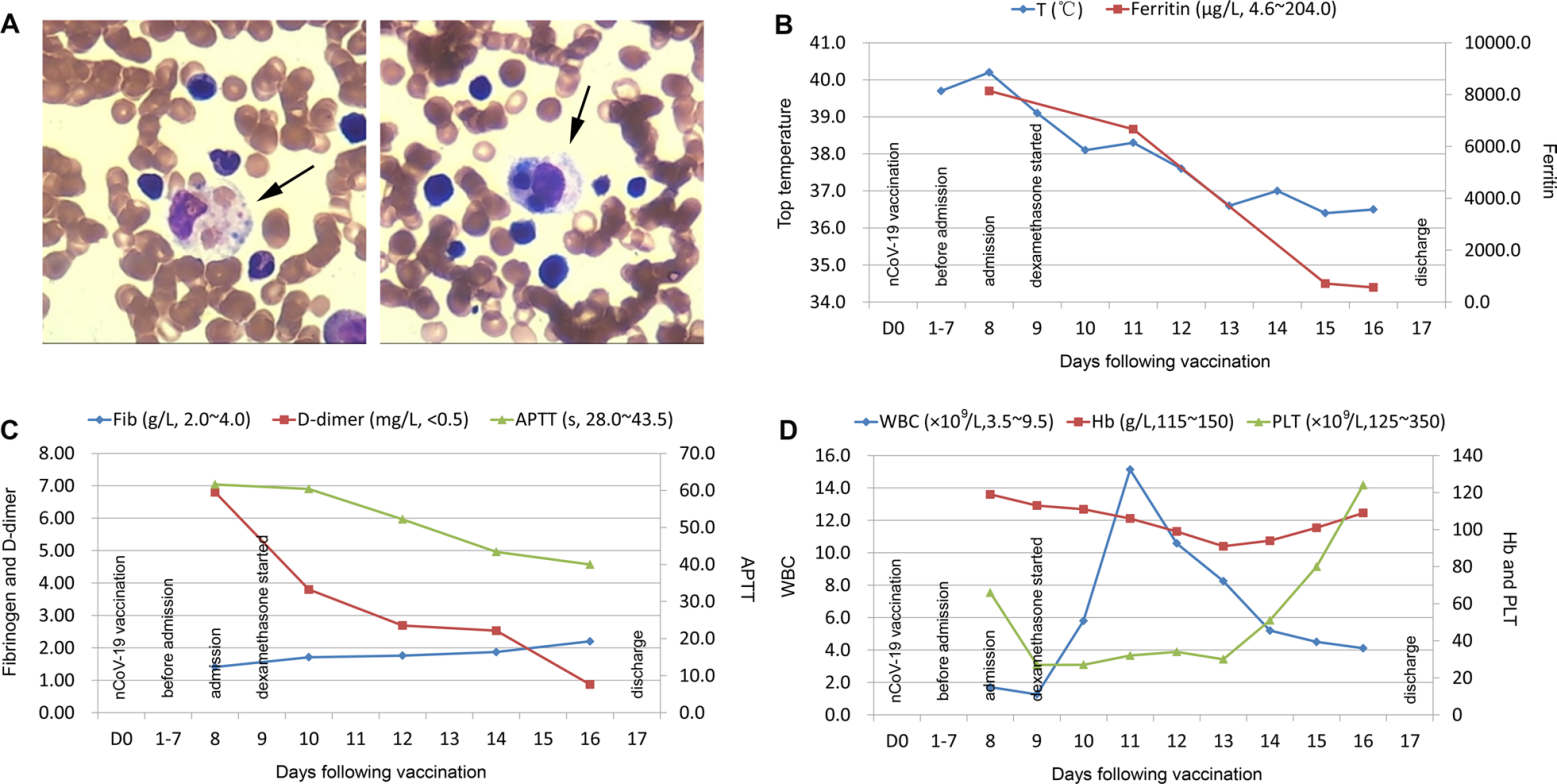
Synopsis of the clinical course. **a** Hemophagocytosis in the bone marrow: the arrows indicate phagocytosis of platelets and erythrocytes by a number of hemophagocytes, **b** Dynamic changes of the body temperature and ferritin level, **c** Dynamic changes of the coagulation parameters, **d** Dynamic changes of the blood cell counts. T: body temperature; Fib: fibrinogen; APTT: activated partial thromboplastin time; WBC: white blood cell; Hb: hemoglobin; PLT: platelet

HLH is a severe and life-threatening hyperinflammatory syndrome caused by aberrant activation of macrophages and cytotoxic T cells. It is characterized by unremitting fever, cytopenia, coagulopathy, hepatic dysfunction, and hypercytokinemia, which may rapidly progress to terminal multiple organ failure, acute respiratory distress syndrome, disseminated intravascular coagulation, and subsequent death [5]. Without early recognition and appropriate treatment, HLH is almost always fatal. In this case, the HLH was well-controlled because of the timely management.

HLH has both primary and secondary forms. Primary HLH is mostly seen in childhood and sometimes even in the elderly with a genetic inheritance, and is caused by various mutations in at least 28 genes involved in the cytolytic pathway proteins such as PRF1, STX11, UNC13D, and STXBP2 [5, 6]. Secondary HLH is a multifactorial disease that can be secondary to infections, hematological malignancies, autoimmune diseases, organ or stem cell transplantation, medications, and most frequently, the combination of these causes. In particular, EBV is the most common trigger for HLH [5]. In this case, we analyzed the known 28 HLH-related genes by next-generation sequencing (Table 1) and did not find a disease-causing mutation. Additionally, this patient had no remarkable medical history, symptoms of acute infection, signs of tumor on the chest-abdomen-pelvis CT scan, detectable autoimmune antibodies, and recent medication intake. Moreover, although both the intracellular and extracellular EBV-DNA were positive, the serological tests (Table 1: EB-VCA IgA−, EB-VCA IgM−, EB-VCA IgG+, EB-VEA IgA−, EB-VEA IgG−, EB-VNA IgG+) indicated that the infection was not a recent event. Therefore, considering the significantly elevated CD8^+^ T cell proportion (71.72%), it is suggested that the HLH in this scenario was induced by acute immunostimulation of COVID-19 vaccination on a chronic EBV infection background.

To our knowledge, this is the first case in the literature to report HLH after receiving the COVID-19 vaccine. Rare vaccination events are important, but do not diminish the well-documented safety profile of the inactivated vaccine against COVID-19, which has been widely administered and shows good immunogenicity, good tolerance, and high efficacy in inducing immune responses against SARS-CoV-2. In addition, this case report should not be seen as a reason to avoid vaccination, since vaccine campaigns are currently still the most promising method to combat the COVID-19 pandemic. Nevertheless, this report indicates that it is crucial to exclude the presence of active EBV infection or other common viruses before COVID-19 vaccination. Patients with underlying conditions should be carefully monitored for any suspicious symptoms and signs following vaccination.

## Data Availability

Not applicable.

## Abbreviations

HLH: Hemophagocytic lymphohistiocytosis
EBV: Epstein–Barr virus
